# Dataset on fuzzy logic based-modelling and optimization of thermophysical properties of nanofluid mixture

**DOI:** 10.1016/j.dib.2019.104547

**Published:** 2019-09-21

**Authors:** Zafar Said, Mohammad Ali Abdelkareem, Hegazy Rezk, Ahmed M. Nassef

**Affiliations:** a University of Sharjah, College of Engineering, Sustainable and Renewable Energy Engineering Department, Sharjah, P. O. Box 27272, United Arab Emirates; b Center for Advanced Materials Research, University of Sharjah, PO Box 27272, Sharjah, United Arab Emirates; c Chemical Engineering Department, Faculty of Engineering, Minia University, Egypt; d College of Engineering at Wadi Addawaser, Prince Sattam Bin Abdulaziz University, Saudi Arabia; e Electrical Engineering Department, Faculty of Engineering, Minia University, Egypt; f Computers and Automatic Control Engineering Department, Faculty of Engineering, Tanta University, Egypt

## Abstract

This article presents the dataset generated during the process of enhancing the thermophysical properties of nanofluid mixture through fuzzy logic based-modelling and particle swarm optimization (PSO) algorithm. The details of fuzzy model and optimization phases were discussed in our work entitled “Fuzzy modeling and optimization for experimental thermophysical properties of water and ethylene glycol mixture for Al_2_O_3_ and TiO_2_ based nanofluids” (Said et al., 2019). In (Said et al., 2019), the detail of the numerical data has not been clearly presented. However, in this article the inputs’ data values for the density, viscosity, and thermal conductivity, used for training and testing of the fuzzy model, have been mentioned which is very essential if the model has to be rebuilt again. Furthermore, the resulting data variation of the cost function for the 100 runs during the optimization process that had not been presented in (Said et al., 2019) is presented in this work. These data sets can be used as references to analyze the data resulting from any other optimization technique. The datasets are provided in the supplementary materials in Tables 1–4

**Table:** Specifications Table

Subject area	*Chemical; modelling*
More specific subject area	*Nanofluid; Artificial Intelligence; Swarm Optimization*
Type of data	*Excel files*
How data was acquired	*The input parameters of PSO from*[2], [3]*. Data of fuzzy model from*[4], [5]*. Afterwards, the numerical simulation was conducted by MATLAB/Simulink software package*
Data format	*Raw, filtered and analyzed*
Experimental factors	*ANFIS of ‘Sugeno-type’ is built based on 3-inputs and one ‘linear’ output. The rule-base is configured using the ‘Subtractive Clustering’ method. The proposed Fuzzy models have 8, 9, 10 fuzzy rules for density, viscosity, and thermal-conductivity models, respectively. Every model is trained with 38 samples for 30 epochs*
Experimental features	*The accuracy of the modeling is confirmed through training the model until a satisfying small testing error is reached*
Data source location	*Wadi Addawaser, Prince Sattam Bin Abdulaziz University, Saudi Arabia*
Data accessibility	*Data are provided in supplementary materials with this article*
Related research article	*Z said, M A Abdelkareem, H Rezk, AM Nassef, Fuzzy modeling and optimization for experimental thermophysical properties of water and ethylene glycol mixture for Al2O3 and TiO*_*2*_*based nanofluids. Powered technology* 2019, 353: 345-358 [1]

**Table:** 

**Value of the data** • The dataset can be reused and extended to build the model with another type of modeling techniques such as Artificial Neural Networks (ANN), Autoregressive-moving average with exogenous (ARMAX), Analysis of variance (ANOVA) or any other technique • These data sets are very useful for making comparisons with other optimization algorithms such as genetic algorithm and cuckoo search optimizer

## 1 Data

This article presents the numerical datasets extracted during the improving process of the thermo-physical properties of nanofluid mixture employing fuzzy logic based-modelling and PSO algorithm. The simulation was carried out using Matlab/Simulink software package on a Core i7 computer with Win10 operating system. Based on the experimental data from [1], an accurate fuzzy model is created to simulate the performance of nanofluid mixture. Tables 1, 2 and 3 (supplementary materials) list the datasets for the density, viscosity, and thermal conductivity, respectively. In every table, the records that show the input-output relationship of the experimental and the resulting fuzzy model outputs for the training and testing data have been presented. The optimal thermophysical properties of nanofluid mixture are identified through PSO algorithm. During the optimization process, the decision variables are represented by three different operating parameters: temperature and the volume fractions of both Al_2_O_3_ and TiO_2_ to enhance the performance of nanofluid mixture which is utilized as a cost function. Due to the stochastic behavior of the swarm optimizers, the optimization results cannot be trusted unless many trials have been done [6], [7]. Therefore, the optimization process was executed for 100 times. Table 4 (supplementary materials) lists the resulting data values of the 100 runs of the optimizer. Each column in the table represents the variation of the cost function for the 50 iterations.

## 2 Experimental design, materials and methods

Two types of nanoparticles used in this study are Al_2_O_3_ with an average particle size of 10 nm and TiO_2_ with an average particle size of 5 nm TiO_2_ nanoparticle with 5 nm. More details about the experimental design and data can be found in [1]. Then, based on these experimental data sets, an accurate fuzzy logic based model is created to simulate the process. Finally, PSO optimizer has been used to identify the optimal operating conditions to enhance the performance of nanofluid mixture. Tables 1–4 of (supplementary materials) show the output of the fuzzy model and the results of optimization process, respectively.
